# Medicinal Plants and Ethnomedicine in Peril: A Case Study from Nepal Himalaya

**DOI:** 10.1155/2014/792789

**Published:** 2014-03-06

**Authors:** Ripu M. Kunwar, Mina Lamichhane Pandey, Laxmi Mahat Kunwar, Ananta Bhandari

**Affiliations:** ^1^Cultural and Spatial Ecology, Department of Geosciences, Florida Atlantic University, 624 NW 13th Street, No. 34, Boca Raton, FL 33486, USA; ^2^Department of Sociology and Anthropology, Tribhuvan University, Kathmandu, Nepal; ^3^WWF Nepal Program, Baluwatar, Kathmandu, Nepal

## Abstract

The impacts of climate change were severe on indigenous medicinal plant species and their dependent communities. The harvesting calendar and picking sites of these species were no longer coinciding and the changes were affecting harvesters' and cultivators' abilities to collect and use those species. Secondary sites: road-heads, wastelands, regenerated forests, and so forth, were being prioritized for collection and the nonindigenous medicinal plant species were being increasingly introduced into the medical repertoire as a substitution and to diversify the local medicinal stock. Acceptance and application of nonindigenous species and sites for livelihood and ethnopharmacopoeias with caution were considered as an important adaptation strategy. Findings on species and site specific accounts urged further researches on medicinal plants, ethnomedicine, and their interrelationship with impacts of climate change.

## 1. Introduction

The rate of warming is increasing in high altitude areas [1–3] where vegetation is rapidly being changed with altitudes [4], offering unique scopes for assessment of climate related impacts [5]. As the warming continues, it is predicted that some irreparable consequences including threats to species, habitats, and distribution range [6, 7] are likely to occur. High altitude forests are more susceptible [8] and the plant species reflect the responses by decreasing species diversity because of the change in plants' functional group or shifting their habitats [9–12]. Individual species either adapt to increased temperatures by modifying their stature and posture [13] or shift towards higher altitudes. Amongst the plants, indigenous plants are expected to be highly susceptible and they are shifting their ranges as a response to climate change [10].

It is hypothesized that as species shift their ranges due to climate change, general and nonindigenous species may fill the vacated niches and outcompete the native species by overwhelming resource exploitation [14]. The native medicinal plants, subsistence produce of the forest dependent communities [15], are particularly threatened by the changes resulting in a direct impact on their dependent communities [16]. Changing ecological and social conditions has transformed and shaped traditional knowledge of medicinal plants to match the new circumstances [17]. The present work was an account to analyze the change of distribution, phenology, and morphology of medicinal plants and their resultant impacts on the mountain communities. We hypothesize that there are changes in medicinal plant distribution, phenology, and population and these medicinal plants dependent human communities are changed and in due course of change, the new plants and sites are accepted as adaptation.

## 2. Materials and Methods

### 2.1. Study Area

**Figure 1:**
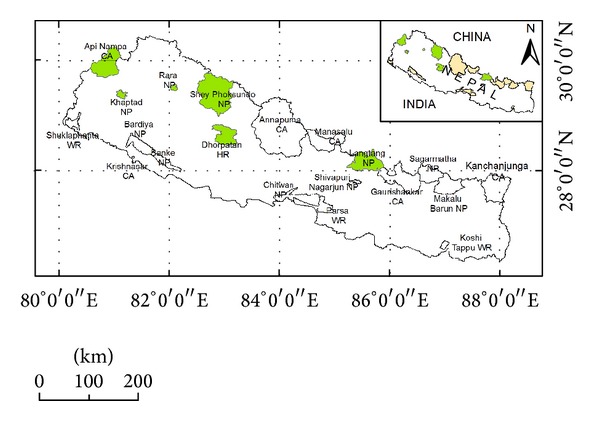
Study area.

A total of six field visits each in one conservation area of Nepal were made. Six different conservation areas (Langtang National Park, Rasuwa district; Shey-Phoksundo National Park, Dolpa district; Rara National Park, Mugu district; Khaptad National Park, Doti district; Dhorpatan Hunting Reserve, Baglung district; and Apinampa conservation area, Darchula district) (Figure 1) were visited between 2007 and 2012. Although some of the conservation areas occupy more than one district, the stated districts herein are meant as sample site.

### 2.2. Participatory Study Methods

Field observations, informal meetings, discussions, and consultations were employed to collect information about folk uses of medicinal plants and local livelihood. In total, two hundred and forty-nine respondents (*N* = 249) took part in eight discussions and ten consultations. A maximum number of discussants (*n* = 76) were from Langtang National Park and the least from Khaptad National Park (*n* = 16). In particular, elderly people, forest guards, and women representing different ethnic groups, castes, and occupations were encouraged to participate. They were asked about the changes, impacts, and adaptation practices of climate change through historical timelines and trend tracking. Their observations, experiences, and expectations were triangulated and used for cross-checking [18].

Matching information between individual surveys and group discussions was taken into account for further analysis. All species encountered during participatory field observations were free-listed and the medicinal plant species were collected during the day and displayed in the evening for discussions. Most of the species were identified in the field using literature of Polunin and Stainton [19] and Stainton [20]. Common species and monospecific genera, those well known by their vernacular names, were used only for discussions and not processed for further identification. The remaining unidentified species were vouchered, identified, and deposited in the National Herbarium and Plant Laboratories (KATH), Godawari, Lalitpur, Nepal. Collection of voucher specimens, along with vernacular names of voucher specimens, was facilitated by eight local assistants. Their assistance was helpful in conducting field level consultations and discussions.

### 2.3. Ecological Study Methods

Rapid assessments and the random field samplings were conducted and the geocoordinates were collected using Garmin eTtrex GPS. Multivariate test was carried out to see the effects of different environmental variables on species richness. The field data of Langtang National Park were grouped in accordance with altitudinal gradients, aspects, and sites and analyzed in the test as a case. Detrended correspondence analysis (DCA) was used to test the heterogeneity of dataset. As the gradient length was 2.567, we used liner redundancy analysis (RDA) method for showing the relationship between species and environment variables following Jongman et al. [21]. Prior informed consents and plant collection permits were granted for participatory and ecological studies. Sometimes plant permit was accounted and do-no-harm plant collection method was approached.

### 2.4. Review

Both the published and unpublished literatures were reviewed and the internet based materials were referenced. Databases of Ethnobotanical Society of Nepal (http://www.eson.org.np/) and Department of Plant Resources (http://www.mapis.org/) and publications of Hara et al. [22–24] were used for species distribution range.

The contribution of herbarium collections to understand local and regional scales of impacts of climate change on ecological processes and species distribution has recently been realized [25–29]. In this study, we reviewed herbarium collections of 19 candidate species: *Abies spectabilis* (Fir), *Betula utilis* (Birch), *Dactylorhiza hatagirea* (Salep orchid), *Ephedra gerardiana* (Joint fir), *Fritillaria cirrhosa *(Fritillaria), *Hippophae salicifolia *(Seabuckthorn), *Juniperus recurva* (Juniper), *Larix himalaica* (Langtang fir), *Lilium nepalense* (Lilium), *Myrica esculenta *(Box myrtle)*, Nardostachys grandiflora* (Spikenard), *Neopicrorhiza scrophulariiflora* (Kutki), *Panax pseudoginseng* (Nepali ginseng), *Podophyllum hexandrum* (May apple), *Rhododendron anthopogon *(Anthopogon),* R. arboreum *(Tree rhododendron),* R. campanulatum* (Blue rhododendron),* Salix calyculata* (Ground salix), and *Taxus wallichiana* (Nepalese yew) housed in KATH. The specimens of samples dated back from 1949 were reviewed and their biogeographic information was computed over time using Canocoo 5.01 [30] and Telwala et al. [31, 32]. Trade data of those 19 species of five consecutive years (2007–2011) available in *Hamro Ban* (official publication of Department of Forests, Government of Nepal) were reviewed. The species used for review were selected based on funding, literature, and frequent citations as highly impacted species due to climate change [33] and the research objectives.

## 3. Results and Discussion

### 3.1. Diversity

A total of 238 useful plant species consisting of 215 genera and 102 families were recorded and among them 192 species were frequently cited as medicinal. Among the medicinal species, 170 species were indigenous and 22 species were nonindigenous. Species are regarded as indigenous at territory, national, and regional level but in the international level they can be considered as nonindigenous [34]. In the present study, we considered that indigenous species are those which grow naturally or they have long been cultured into an area after some sorts of human modifications. Globally, native or nonnative status is generally determined by one (or both) of two concepts: (1) presence in an area before an arbitrary cut-off date imparts native status and (2) human-mediated movement of individuals results in nonnative status [35].

### 3.2. Use

The use of high percentages (80) of indigenous species was an indicative of ancient healing tradition but remained somewhat diffused because of the application of nonindigenous ones. The use of nonindigenous species in local traditional medicine was similar to the findings of a number of other ethnobotanical studies [36–38], emphasizing the need for more scrutiny and efforts to record and maintain traditional knowledge. As elsewhere, adoption of nonindigenous species was increasing may be seen as a way to reshape and revitalize traditional practices, which in many cases provide an important alternative to the health care services [39]. A larger number of indigenous and nonindigenous species and pharmacopoeias were embraced due to increasing health care demand and the wider range of illnesses [40–43]. Ethnomedicinal studies, therefore, have shown the relevance of nonindigenous species as an asset for local medicinal stock [44].

### 3.3. Distribution

Distribution of medicinal plant species was species specific. Tree species *B. utilis* (Birch), *A. spectabilis* (Fir), and *J. recurva* (Juniper) and understorey *N. grandiflora *(Spikenard) and *D. hatagirea *(Salep orchid), were specific to their restricted distribution resulting in strenuous collection of their produce. *Betula *and *Dactylorhiza* were more susceptible due to their small population sizes (0.0058/m^2^, 0.35/m^2^ resp.) and limited suitable habitats [45]. Their distribution was restrained by outcompetition of *R. campanulatum, Cotoneaster species*, and* A. spectabilis* resulting in likeliness of pushing *Betula *and *Dactylorhiza* off the mountain tops [46, 47].

**Figure 2:**
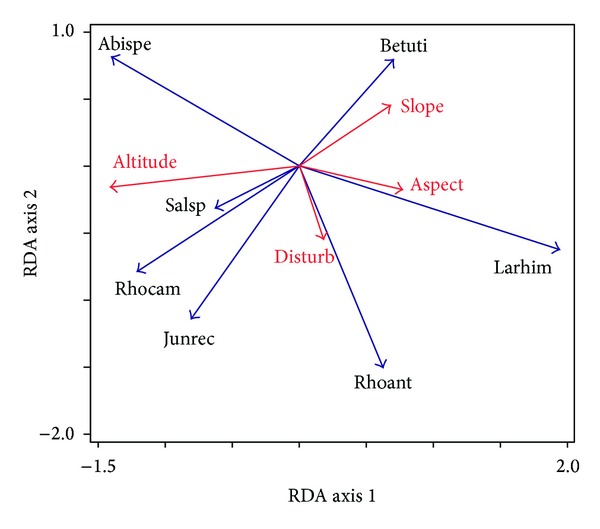
RDA biplot showing composition of significant environmental variables that influence the distribution of plant species in Langtang National Park, Central Nepal. Species abbreviated in figure are as follows Abispe = *Abies spectabilis*, Betuti = *Betula utilis*, Junrec = *Juniperus recurva*, Larhim = *Larix himalaica*, Rhoant = *Rhododendron anthopogon*, Rhocam = *R. campanulatum*, and Salsp = Salix species.

The biogeographic information of plant herbarium showed the higher altitudes of collections over time. The result was consistent with the earlier observations as found on *F. cirrhosa* and *H. salicifolia* [48]. The distribution records of species from lower altitudes in earlier days and the subsequent records from successive higher altitudes were corroborating with distribution upshifts. We found the upshifts of *L. himalaica *and *P. roxburghii* 4 m per year and that of *R. arboreum* as 0.88 m per year. Upshift of *A. spectabilis* observed as 2.5 per year in Langtang, Central Nepal, substantiated the earlier findings [49–51] but, in general, vegetation upshift in response to climate change ranges within 1-2 m per year [52]. Change in distribution of useful species and primary habitats showed the importance of the use of secondary forests, nonnative species, and underutilized species [53]. The change in distribution was consistent to the findings of disturbance gradients analysis (Figure 2). Out of four different environmental variables computed, only altitude and disturbance were significant for the change of distribution of plant species. First axis explains 15.47% and the second axis explains 2.36% of the total variation in the dataset (Figure 2). Altitude possessed the positive correlation with *R. campanulatum*, *J. recurva*, and *Salix* species whereas *R. anthopogon* and *L. himalaica* were influenced by disturbance. West facing slope revealed strong affinity to the regeneration and seedling growth of *J. recurva*.

Because of the changes in distribution and upshifts, some of the picking sites of medicinal plant species were no longer coinciding and the abilities of the harvesters' to collect and use plants were being affected. The picking sites of medicinal plants were particularly dissenting in conservation areas such as Khaptad National Park and Rara National Park at lower elevation and the secondary sites were increasingly sought. At lower elevation of study sites invasion of nonnative plants *Ageratum conyzoides*, *Bidens pilosa*, *Eupatorium odoratum*, *Lantana camara*, *Parthenium hysterophorus*, and so forth was frequent as found in other parts of the country [54, 55]. Former two species were ranged up to 3000 m and introduced at lower elevations of Langtang National Park and Shey-Phoksundo National Park. The frequent infestations were seen along the roads, wastelands, fallow lands, and grazing sites. Species *Taraxacum officinale* was sometimes found at 3000 m or above of Dhorpatan Hunting Reserve and Rara National Park; however these conservation areas were not yet faced with problematic intrusions by alien invasive species. The invasion was also complemented by outmigration of people. The outmigration laid the agricultural field fallow, decreased agricultural productivity, and contributed to the deficit of human resources for management, aiding habitat deterioration, and invasion [56]. As a consequence, diversity and distribution of indigenous medicinal plant species were increasingly imperiled and livelihood was compounded.

### 3.4. Morphology and Phenology

Small, stunted, and multistemmed individuals as adaptive features of trees were seen at higher elevations in response to climate change, yet the individuals were in isolation. *Abies* trees with smaller height and low canopy (shrub forest and groove) were observed at higher elevations to resilient the climate change. Clonal growth and high coppicing properties as evident in *R. anthopogon* and peeling bark in *R. campanulatum* were also considered as adaptive features. Early leaf emergence was observed in *S. sikkimensis* whereas advance flower initiation was seen in *L. himalaica*. These advance adjustments of their phenophases were made by plants in response to climate change and earlier spring [57]. Early bud burst and flowering based on indigenous knowledge regarding climate change impacts were earlier evidenced [58, 59]. The shift in phenophases that seems to be the immediate impact of warming on the physiology of species [60] is bound to prolong the total growth duration of the species, which is regarded as benefit to the plant productivity. Early flowering of *R. arboreum* and *R. campanulatum* was seen but that of *R. anthopogon* could not be observed, so it was difficult to conclude how the climate change affects plant phenology because the changes are species and microclimate specific. Shifting phenologies and distribution may seem to be of little importance at first glance [16], but they have the potential to cause great challenges to species' survival and people's livelihood.

Besides the changes in phenology, morphology, and distribution of plants, the secondary metabolites and other compounds of Plants-produce which usually value for therapeutic properties [61] are expected to change. Generally when plants are stressed, secondary metabolite production may change as the growth is often inhibited [11, 14, 16, 46, 62]. However, the change on secondary chemical production in plants is largely unclear [63]. In either change, the plants' decade-long therapeutic potential for human health benefits may no longer retain, resulting in threatened ethnomedicine.

### 3.5. Medicinal Plants and Livelihood

The result supported that the longer the history of contact of a community with nature, the higher the number of medicinal plants used, as well as the higher the number of ailments treated [43, 64, 65]. The earliest written records of plants used as medicine in the Nepal Himalaya are found in the 6,500-year-old texts of the *Rigveda* [66], 4000-year-old text of the *Atharvaveda*, and 2500–3000-year-old texts of the *Ayurveda* [67, 68]. Catalogues have recorded about 2,400 (33% of country's flowering plants) useful medicinal and aromatic plants in Nepal [69] and their importance in alleviating human suffering [53, 70]. Of 192 plants used for ethnomedicine, most of them (169 species) were used for more than one ailment. Species *Aegle marmelos*, *Cissampelos pareira*, and *Terminalia bellirica* each were used for treatment of six ailments. Species used for treatment of five ailments were *Acorus calamus*, *Bergenia ciliata*, and* Ziziphus mauritiana*. A total of 66 ailments were treated using folk lore and among them, dysentery, diarrhea, and skin problems were the most treated, respectively, by 24, 22, and 22 species. A large number of botanicals used in ethnomedicine were characteristics of medicinal plant species diversity. The extensive usage of medicinal plants for ethnomedicine showed that it was not merely a medical system but a part of culture. Again, multiple uses of a plant gave us idea that the area was equally rich in botanical knowledge.

Species *A. spectabilis*, *Paris polyphylla*, *O. sinensis*, and *Z. armatum *common in study area and widespread in use were in great peril because of multiple uses. The result also supported the notion that the more versatile a plant, the more widespread its usefulness [71].

*A. spectabilis*, *L. himalaica*, *P. roxburghii*, *and R. arboreum* were pushed off the mountain tops and they were also overexploited in medicinal and cultural usage. *Abies* leaf needles were sniffed for cough and cold. *Abies* poles were used for mounting flags. Shoots were heavily logged for furniture and agriculture implements. Survival of species with multiple uses was also compounded because of their versatile uses: they were fetching higher prices in markets, useful as spices, condiments, medicinal, and tonic. Among the 19 studied species, eight species (*B. utilis*, *E. gerardiana*, *J. recurva*, *L. nepalense*, *N. grandiflora*, *N. scrophulariiflora*, *P. hexandrum*, and* R. anthopogon*) were the most in trade nonetheless their volume in trade was significantly plummeted. The total traded volume of these species was 753 tons in 2007 and only about 100 tons in 2011. The annual Nepalese medicinal plant trade of total species varied from 480 to 2,500 tons over time [68]. In the changed contexts, livelihood was more vulnerable and the alternatives were frequently sought. Therefore the application of new species and sites was feasible and in due course the usage becomes an asset of adaptive knowledge.

Livelihood diversification (subsistence agriculture to commercial farming and ecotourism), crop substitution (seeking new crops and varieties), changing calendars (pre- or postfarming), off-farm employment (porter, trekking, and hotel), seasonal migration, and so forth were dominant traditional adaptation strategies for climate change however they were varied in sites. Off-farm employment was increasingly adopted in Langtang National Park where there was a huge impact of visitors. Intensive crop and farming related strategies were frequent in study districts of Apinampa Conservation area and Dhorpatan Hunting Reserve where folks have long been involving in subsistence agriculture and they have not been greatly intruded by visitors, thanks to the rugged terrain and physiography of these conservation areas. Seasonal migration, a traditional adaptation strategy and common in Shey-Phoksundo National park, offers scopes for sharing ideas and goods. Folks were intending to diversify the livelihood in Shey-Phoksundo, Rara, and Khaptad National Parks where there were mixed impacts of tourism, commercial farming, and modernization. As a result, acceptance and application of new species and sites for livelihood were considered important for adaptation. New sites, previously neglected such as road sides, disturbed forests, forest fringes, and agricultural ecotones, were increasingly being browsed attributed to the business of local communities and accepting the sites as adaptation assets. Again, knowledge, cultivation, and maintenance of these species within rural communities were decreasing caused by the modernization processes, such as acculturation. Loss of traditional knowledge and even the physical annihilation of indigenous groups not only impede the search for new drug plants but also handicap the efforts to conservation [72].

Present study found 22 nonindigenous species (*Acmella calva*, *Adiantum capillus-veneris*, *Ageratum conyzoides*, *Aloe vera*, *Angelica archangelica*, *Cirsium verutum*, *Cissampelos pareira*, *Drymaria cordata*, *Eclipta prostrata*, *Elephantopus scaber*, *Entada phaseoloides*, *Evolvulus alsinoides*, *Holarrhena pubescens*, *Ipomoea carnea*, *Jatropha curcas*, *Mimosa pudica*, *Plantago major*, *Plumeria rubra*, *Psidium guajava*, *Ricinus communis*, *Smilax aspera*, *and Xanthium strumarium*) and they have long been cultured into ethnopharmacopoeias of Nepal Himalaya. We can claim that the culture of nonindigenous species on ethnopharmacopoeias is mostly as a substitution because they were introduced over time, corroborated to the earlier findings [70–74]. Nonnative species have been incorporated into *materia medica* from around the world [44]; however their importance has not been credited [75]. The entrance of nonindigenous plant species in a pharmacopoeia is a natural and evolutionary phenomenon and we need to be cautious when employing them into medical repertoire and attributing their values [76] as the introduction of the nonindigenous species can be both boon and bane to the society [77].

## 4. Conclusion

Adjustments in distribution, phenology, and population of plants jeopardized the species survival and livelihood of mountain communities. Tree species *A. spectabilis*, *B. utilis*, and *J. recurva* and understorey species *N. grandiflora* and *D. hatagirea* were mainly threatened due to the population size and site specific distribution. *A. spectabilis*, *F. cirrhosa*, *H. salicifolia*, *L. himalaica*, *P. roxburghii*, and *R. arboreum* did reveal not only the upshifts but also the fact that their distribution was governed by altitude and disturbance gradients. Because of the changes in distribution and upshifts, some of the original picking sites of these species were dissented and the harvesters' abilities to collect and use those species were affected. We found that the more versatile a plant is, the more widespread its usefulness is and the more usefulness a plant has, the more overexploited and endangered it is likely to be. Species *A. spectabilis*, *Acorus calamus*, *Aegle marmelos*, *B. ciliata*, *C. pareira*, *P. polyphylla, O. sinensis*, *T. bellirica*, *Z. armatum*, and* Z. mauritiana* were widespread in use and in great peril because of their multiple uses. We found 22 nonindigenous species that have been introduced into ethnopharmacopoeias of Nepal Himalaya. Acceptance of nonindigenous species and sites for livelihood and medical repertoire as a substitution was considered as an adaptation but we should be cautious when attributing their values.
